# AI Use or Non-use by First-Year Medical Students: A Qualitative Study of Perspectives, Usage, and Recommendations

**DOI:** 10.12688/mep.21319.2

**Published:** 2026-02-18

**Authors:** Jennifer Simoni, Elisa Mengual, Ines Aschenbrenner-Noriega, Clara Monforte-Martínez, Marta Pérez-Merino, Gabriela Sawczyn, Rocío Zurita, José Luis Pereira

**Affiliations:** 1 Unidad de Educacion Medica, Universidad de Navarra Facultad de Medicina, Pamplona, Navarre, 31071, Spain; 2 Dept. de Patologia, Anatomia y Fisiologia, Universidad de Navarra Facultad de Medicina, Pamplona, Navarre, 31071, Spain; 3 Universidad de Navarra Instituto de Cultural y Sociedad, Pamplona, Navarre, 31071, Spain

**Keywords:** Undergraduate Medical Education, Curriculum Development, Thematic Analysis, Student Perspectives, AI Literacy, Competency Framework, Ethical Considerations, Co-design

## Abstract

Background Artificial Intelligence (AI) is reshaping healthcare and medical education, with growing calls to embed it in medical curricula. However, evidence on first-year medical students, perceived benefits and limitations of AI, and views on ethics and professionalism is limited. Methods A qualitative study was conducted using semi-structured interviews to explore the experiences, attitudes, and perceptions of first-year students regarding AI. Convenience sampling yielded the participant cohort. Recruitment and analysis continued until thematic saturation was achieved. Transcripts were coded iteratively using NVivo software, and a reflexive thematic analysis was undertaken. Results Twenty participants were interviewed; 18 were AI users, to varying degrees, and two were non-users. Seven themes emerged: How AI is used; Benefits; Concerns and limitations; Ethical considerations; Advice for peers and professors; Attitudes toward and understanding of AI; and Participation in the project. AI users cited motivations like efficiency, personalization, and support. Benefits included faster access to information, organized content, and tailored explanations. Concerns included AI reliability, over-reliance, and ethical misuse, such as plagiarism. Most supported the inclusion of AI literacy in curricula for responsible, practical, and critical use of AI. Participants with AI literacy demonstrated a deeper understanding of AI. Conclusions We found that students in the medical school we studied are early adopters of AI, using it in various ways, and wish to utilize it effectively and ethically. The findings of this study align with other studies in other jurisdictions that call for early AI literacy.

**List of abbreviations**
AI: Artificial Intelligence
*AI*
: *UNAV Student Collaborative Team* – *Artificial Intelligence Universidad de Navarra Student Collaborative Team*
LLM: Large language modeling
UGME: Undergraduate Medical Education
UNAV: University of Navarra

## Background

The use of Artificial Intelligence (AI) in medical education has emerged as a groundbreaking and rapidly evolving phenomenon that educators, learners, and institutions must adapt to judiciously and keep pace with. ^1^^,^^2^ Artificial intelligence encompasses several distinct and continually evolving subfields and technologies, one of which is large language modeling (LLM), which is finding various applications in medical education. ^3^ The release of ChatGPT in November 2022 has, for example, become a disruptive innovation, with medical education experts expressing mixed sentiments about it but broadly recognizing its potential benefits. ^4^ ChatGPT and other LLM technologies are giving students, educators, and institutions direct access to powerful AI capabilities.

There is general agreement that the use of AI technologies in education and the medical field is already widespread. However, it is still in the early stages of integration into medical curricula. ^5^ Given that these technologies can potentially enhance, and perhaps even replace, some medical skills and generate new clinician roles, ^6^ there are growing calls to make AI literacy a core competency for medical students. ^7^^–^^10^

With or without guidance, medical students are already using AI technologies in their undergraduate education. ^1^ Studies of Canadian ^11^ and German medical students, ^12^^,^^13^ using mixed research methods, have reported inadequate training in this area and the need to incorporate AI in medical curricula. A survey-based study among Australian medical students came to the same conclusions. ^14^ Instruments, such as the Medical Artificial Intelligence Readiness Scale (MAIRS), are being deployed to evaluate and monitor the readiness and literacy levels of medical students regarding AI technologies and applications. ^15^^–^^19^

While past studies have provided valuable insights into the use of AI by medical students, including the why, when, and how they use it, as well as their perceptions of the ethical and professional implications of its use, further exploration is needed. ^1^^,^^20^ Most studies have included students in all years or have focused on senior students only. ^21^^–^^23^ In contrast, there is a lack of studies on recent and current cohorts of first-year medical students ^6^ (2023–2024), who, as early adopters of new LLM technologies, including ChatGPT, represent a unique group. Unlike past and current senior students, who may have developed more structured study habits outside of AI and LLM everyday use, the 2023 and 2024 students are still in the early stages of forming their learning strategies. Moreover, as early adopters and as the next generation of clinicians, they could inform new AI-related curricula. ^24^

To integrate AI-related literacy and competencies into its undergraduate medical curriculum, and to nurture scholarship, a team of students, known as the *Artificial Intelligence Universidad de Navarra Student Collaborative Team* ( *AI UNAV Student Collaborative Team*) and three professors at the University of Navarra in Spain, are undertaking an initiative with several components. ^25^ Some students are interested in AI, while others are more interested in developing scholarship skills, as one of the goals of the overall initiative is to nurture scholarship among students with a special interest in this area. These component of the initiative include: 1) the present study, (reported herein); 2) a scoping review of the literature on AI in medical education (from 2022 to 2024) ^26^; 3) a Delphi process to identify the competencies and learning objectives to include in the medical curricula (submitted for publication); and 4) student-driven guidelines on the practical, professional, and ethical use of AI to support learning. Additionally, the initiative aimed to provide students with opportunities to develop their CanMEDS Scholarship roles, ^27^ including participation in curriculum development and research. Thus, a co-design approach was incorporated, involving close collaboration between faculty and students. ^28^^–^^30^

The study described herein aimed to explore, in more depth, why and how first-year medical students currently use AI, or don’t use it in their medical training, including their motivations for using it or not using it, methods of use, and overall experiences.

## Methods

### Overall study design

A qualitative research approach, utilizing semi-structured interviews, was employed. This approach was chosen because the study’s goals were to gather rich, in-depth data and understand a complex, nuanced, and emerging phenomenon. ^31^ We adhered to the Standards for Reporting Qualitative Research (SRQR) for reporting standards. ^32^

Research was conducted by a team of two faculty professors with qualitative research experience (JS, JP) and five students from the *AI UNAV Student Collaborative Team.* The two professors trained and supervised these five students in qualitative research methods, including interview skills, data coding, and thematic analysis. ^33^

### Participants and setting

Participants were recruited using convenience sampling, with an open invitation extended via email to all first-year medical students at the University of Navarra. We used a combination of convenience and purposive sampling to ensure representation by students with various levels of AI usage, from extensive use to non-use. Interviews were conducted at the end of their first academic year (May–June 2024) when students had a full year’s experience. Participation was voluntary, and students provided informed consent. Students were drawn from the two tracks in the first year: the Spanish track and the international track. In the latter classes, which are primarily given in English, most students preferred to participate in English. Recruitment continued until saturation was reached. The five students who were part of the research team in this qualitative study did not participate as interviewees.

### Data collection

A semi-structured interview guide was developed, with guiding questions aligned with the study’s research questions (see extended data). Participants were asked a series of open-ended questions to explore their experiences and perspectives. These questions included whether they were using AI and their reasons for doing so (or not), how and when they used AI, and their overall experiences with it. Students were also asked to reflect on the advantages and disadvantages they encountered, as well as any concerns they had regarding its use. Additionally, they were invited to offer advice to their peers on how to use AI effectively and to suggest ways professors and faculty might incorporate AI into teaching, including recommendations for integrating AI into the curriculum. Finally, participants were allowed to share any other thoughts or insights on the topic.

The guide was first piloted with three students to ensure clarity and relevance. After conducting the first three interviews, a preliminary analysis was performed, and minor modifications were made to the guide to clarify, expand, and address additional concepts obtained from the initial interviews.

Three members of the research team conducted each interview: one professor and two students. Initially, the professors took on the lead interviewer role, and later, the research team students assumed more of this role as they gained confidence, always under the supervision of one of the professors. Interviews were conducted in person. The interviews lasted approximately 30 minutes each, were audio-recorded with the participant’s consent, and then transcribed verbatim. Interviews were conducted in Spanish or English, depending on the participant’s preference, and transcribed in the original language. ^34^ The transcripts were de-identified by the lead researcher (JS), assigned a number, and then made available to the whole team for analysis.

We purposively chose not to define “AI” to allow students to discuss AI in a manner that reflected their personal experiences and perceptions. Our goal was to capture the full range of student interactions with AI tools, regardless of the specific technologies or platforms they used.

### Data analysis

The initial analyses were conducted using printed copies of the transcripts to help students become acquainted with the coding process. All coding was done in the original language, English or Spanish, but codes were maintained in English for consistency. ^34^ All transcripts were transferred to NVivo 14 software to facilitate data management and analysis across all transcripts.

The data were analyzed using thematic analysis. ^35^ First familiarization of the data occurred by reading the transcripts, followed by coding, categorizing the codes into groups, and identifying recurring themes.

An iterative process was undertaken where interviews were conducted in three batches, and coding was done after each batch. The coding process began with the first three interviews being independently coded by each research team member, followed by a joint session where the team members reached a consensus on initial codes. This collaborative coding process also promoted a shared understanding of the data and provided additional insights. Coding and re-coding continued iteratively in batches, adding new codes and themes as additional interviews were analyzed. By the ninth interview, no further code modifications were required. The two faculty team members independently coded the final eleven interviews using the established codebook and held regular meetings to reach consensus on the coding. These were presented to the research team regularly so that the whole team could participate in coding checks and resolve any discrepancies until consensus was reached. We started from a position that all input was respected. When there was disagreement, a discussion was held to ensure the different views were heard and understood. From that discussion, we reached a consensus solution that reflected their thinking, or, if their perspective changed, we adjusted accordingly.

The research team collectively intentionally repeatedly asked itself whether it had fully coded all relevant data, whether the codes were applicable and appropriate, whether it had fully and appropriately grouped related codes into potential themes and whether the themes identified were representative of the data, whether the themes had been fully refined to ensure coherence and relevance, whether the themes were each supported by sufficient data excerpts, and that no key aspects of the data (and codes and themes) had been overlooked. This aligns with a checklist recently described by Ahmed et al. ^36^

We intentionally applied reflexivity to address potential biases, given that the research team included members with their own personal views on and varying levels of use of AI, and it included both professors and students, introducing potential power dynamics. Moreover, we were cognizant that some of the interviews were undertaken by students who were participants’ class peers. ^37^^,^^38^ We acknowledged the inherent power dynamics within the team, particularly the possibility that students might feel hesitant to voice their opinions during interviews or data analysis due to the hierarchical relationship with professors. To mitigate this, we fostered an open and collaborative environment where all contributions were valued equally. Throughout the coding and analysis process, the research team -professors and student researchers - actively reflected on their own interpretations and consistently sought input from student researchers. ^39^

Lastly, a bilingual researcher, who is a native Spanish speaker, translated the Spanish transcripts into English to ensure the authenticity of the translation and verify cultural nuances. All quotes included are in English. ^40^ A letter after the participant’s number indicates whether the original language was Spanish (S) or English (E).

### Ethical considerations

The University of Navarra Research Ethics Committee reviewed and approved the study protocol (2024.084 mod1). The purpose of the study was explained to the participants, and written informed consent was obtained at the beginning of each interview. Participants had the right to withdraw at any time. Each interviewee was assigned an identification number for confidentiality purposes, and all interviews were de-identified after transcription. The research team compiled and analyzed de-identified transcripts.

## Results

Twenty students were interviewed. Of the 20 students (12 female and 8 male), 10 were part of *AI UNAV Student Collaborative Team*, and the other 10 were not involved in the AI initiative. Eighteen used AI, with a broad scope of frequency across them, and two were non-users.

Seven main themes were identified. These included A) How AI is used; B) Benefits of AI; C) Concerns and limitations of AI in medical education; D) Ethics of use of AI in learning; E) Advice on AI use in medical education; F) Attitudes toward and understanding of AI; and G) Participation in the project. Table 1 summarizes the themes, their descriptions, their respective subthemes, and illustrative quotes. There is an overlap between some themes, such as the Use of AI (and its motivations and rationale for use) and perceived Benefits.

**Table 1.** Themes, subthemes, and descriptions of themes with illustrative quotes.

Themes	Theme Description	Subthemes	Illustrative Quotes
**How AI is used**	Participants described the workload in medical school as extensive, burdensome, and time-consuming, leading to a perceived need for additional support and strategies to facilitate their learning. Participants described several ways in which AI helped them achieve this goal.	Rationale and Goal (in learning) Introduction to AI Type of AI tools used Strategies when using AI Frequency of use	*“With the pace that we have with studying, it's not always easy to go and open a book and spend a few minutes trying to find the pages or go and flip through the Internet trying to find the answer. So that just makes the process really quick and easy.” (P13E)*
**Benefits of AI**	Students reported that AI improves their ability to manage the demanding workload of medical school by providing concise, well-organized information, instant feedback, and provides a non-judgmental environment for exploring concepts. AI was also described as a supplemental tool that fills gaps in their education when other resources, such as professors, are not readily available.	Efficiency Organized and concise information Personalization Non-Judgmental Learning	*“Mostly the speed in getting a response. In a book, you have to read and analyze yourself to get the information, but with* ChatGPT *, it takes much less time, and the response is more specific and concrete to what I need.” (P4S)* *“It’s convenient, concise, and you can ask anything. Your professor might think you’re stupid, but the AI won’t judge you.” (P20E)*
**Concerns and limitations of AI**	Students expressed significant concerns and identified limitations. These included issues related to the reliability of AI-generated information, its potential to stifle creativity, and the risk of over-reliance, which could hinder the development of critical thinking and independent learning skills.	Reliability Too general or too specific Stifling Creativity and Work Ethic Overreliance and Excessive Use	*“The negatives are that it's not always right and it can influence your learning and understanding,” (P19E)* *“It has fooled me a few times with the wrong answers.” (P20E)* *“I just want to have a specific answer and then it tells me this long, long kind of a story. I don’t want to read it all.” (P1E)*
**Ethics of AI**	Ethical considerations were a significant concern among students, particularly regarding plagiarism, work ethic, and preserving human qualities such as empathy and compassion in medical practice. While students acknowledged AI’s potential to support learning and decision-making, they emphasized the importance of maintaining human oversight and responsibility in educational and clinical settings.	Plagiarism Work Ethic Loss of Empathy and Compassion (Human Touch) Human Responsibility	*“There is, you know, plagiarism. It would be easier to pass some exams depending on where you are if you can use it. You can use it to cheat, and then you're going to treat people with questionable knowledge.” (P18E)* *“Work ethic-wise it definitely contributes to laziness.” (P13E)* *“Doctors should ultimately be making their own choices, and if they decide to take the opinion of an AI as part of that, they still hold responsibility for whatever action they take, not the artificial intelligence” (P7E)*
**Advice on AI Use in Medical Education**	Students offered practical advice to their peers, professors, and curriculum developers regarding AI's responsible and effective use in medical education. Their advice emphasized the importance of using AI as a supportive tool, maintaining academic integrity, and incorporating AI instruction into the curriculum in a structured and meaningful way.	Advice for Peers Advice for Professors Advice for the Curriculum Development	*“I would recommend you and your teacher being the primary tool and your notes in class, and then going to* ChatGPT *or any other AI resource as a secondary tool.” (P13E)* *“Professors should use AI in ways they would want others to use it. They need to be aware of the impact of their actions and use AI responsibly. For example, if students don’t understand a topic, professors can use AI to create additional study materials that could be beneficial.” (P9S)* *“I would integrate it into classes because it's going to be part of our lives. The sooner we learn the advantages, disadvantages, and how to use it properly, the better.“(P4S)*
**Attitudes toward and understanding of AI**	This theme explores students' sentiments toward AI, reflecting a spectrum of attitudes from enthusiasm and curiosity to skepticism and uncertainty about its role in medical education and future clinical practice. Additionally, it examines students’ varying levels of understanding and familiarity with AI tools.		*“In general, in today's society, people don’t want to think. People want things instantly without spending time and energy doing things.” (P1E)* *“It's definitely going to change the profession. Some people say it's going to replace doctors, but I don't believe it. It's hard to predict how medicine is going to be influenced exactly, but I guess over the years, the influence of AI is going to increase in all fields.” (P18E)*
**Participation in Project**	This theme highlights students' experiences and reflections in the *AI UNAV Collaborative Student Team.* Their involvement in the project enhanced their understanding of AI and influenced their behavior and attitudes toward its use.		*“Since I've started to be involved in this project about AI, I've started to use AI less because it made me more cautious about the potential mistakes that AI makes. So, in reality, my use of AI has decreased” (P3E)*

### How AI is used

This theme explores why, how, and when participants use AI to support their learning. Five subthemes were identified: a) Rationale and Goal; b) Introduction to AI; c) Type of AI tool used; d) Strategies when using AI; and e) Frequency of use.

***Rationale and Goal (in learning).*** Participants described the workload in medical school as extensive, burdensome, and time-consuming, leading to a perceived need for additional support and strategies to facilitate their learning. Participants described several ways in which AI helped them achieve this goal. Providing efficiency was foremost among these and accomplished in several ways. Some used their preferred AI platform – often ChatGPT – as a quick search engine when they want further explanation of terms or concepts they have read about or heard in the classroom. Others, on the other hand, began studying a topic by first visiting an AI platform and prompting it to provide an overview. They then used that information to explore further details in textbooks, online resources, and classroom materials.

• “With the pace that we have with studying, it’s not always easy to go and open a book and spend a few minutes trying to find the pages or go and flip through the Internet trying to find the answer. So that just makes the process really quick and easy.” (P13E)
• “If there’s a very large text which I don’t really understand, maybe I would ask for a summary” (P6E)

Participants liked using AI as a personal tutor.

• “I basically use artificial intelligence like a Google search engine, but when Google doesn’t give me what I’m looking for or doesn’t explain it in a way I understand, I use artificial intelligence to understand it on my own before asking a classmate. It’s like having another classmate who explains a lesson to you.” (P2S)

Others asked AI to critique their notes and to provide personal feedback.

• “When I do my notes, I make a perfect paragraph of the summary. After having this whole dialogue, I would be like, “OK, is this right?” Then I paste my summary, and it can be like, “Yes, it’s the perfect summary,” or “Yes, but let me clarify some things.” Then it rewrites it and adds a little bit, and then I can choose to add that to my notes or not.” (P20E)

Interestingly, some students use AI to prepare more efficiently for examinations by prompting it to create examination questions, including multiple-choice tests, and provide correct answers related to a topic they are studying.

• “Sometimes I copy and paste information from notes and put a command like “make 10 university-level questions from this text with A, B, C, D options,” and it makes them for me.” (P18E)

***Introduction to AI.*** Most commonly, participants reported being introduced to AI by their peers. Since ChatGPT (the most mentioned AI platform among the participants) was released during their last year of high school (2022), they may not have been introduced to AI before medical school.

• *“I saw a classmate using it; she recommended it to me, and we tried it out right then for a doubt I had at that moment. She said, “I don’t know, try asking* ChatGPT *these specific questions,” and I started using it.” (P12S)*

Some were led to use AI when they heard that professors were using it to help them develop examination questions.

• *“It was at the end of the first semester when a professor told us he made exam questions using* ChatGPT *. So, I thought to myself, OK, if he does it, I can also do that. That’s when I started using it.” (P16E)*

***Type of AI tools used.*** The participants used various AI platforms and tools. Generative AI platforms, especially ChatGPT, were the most mentioned. Some learners were aware of and used other platforms, sometimes simply due to convenience, as they were already installed on their computers. One student with a special interest in AI was developing their own LLM.

• *“I mainly use* ChatGPT *, but I also use translation tools. My sister recommended Bing, but I don’t use it as much.* ChatGPT *is like the Google of search engines for me.” (P2S)*
• “I had also used tools like DeepL for translating texts, which use artificial intelligence.” (P2S)
• “Mainly because Bing came with my computer” (P14S)

***Strategies when using AI.*** Participants employed various techniques to engage AI systems to support their learning. Effective prompt writing using LLMs was frequently mentioned. The need for an iterative process and fine-tuning the prompts was described. Some students admitted that they had not invested much effort in optimizing their use of AI.

• “Over time, I’ve learned to ask more specific questions and provide clearer prompts. This has significantly improved the quality of the responses I receive.” (P9S)
• “I just write really basic prompts like, ‘Please explain to me this for a medical student in detail.’ And if I don’t understand it, I’ll ask it, ‘Please explain it to me in a simpler way,’ or ‘I did not understand this. Explain it to me another way.’” (P13E)
• “No, I’m not that committed to it.”. (P18E)

***Frequency of use.*** The frequency of use to support learning varied considerably across participants. Some were frequent users, while others used it less frequently or seldom.

• “Two times a day if I’m studying the whole day.” (P20E)
• “Not very often, maybe once a week. It’s only for exceptional cases when nothing else has been clear because I know there can be errors, so I use it as a last resort.” (P4S)

Participants described the difficulty of the course load at the beginning of medical school and their unfamiliarity with the material, which dictated their more frequent use.

• “At the beginning, I used it a lot because I needed it. I had no idea about many things. Recently, I’ve stopped using AI as much because I’ve learned how to find information in books or slides provided by the teacher.” (P5E)

Others have discovered AI’s benefits and have created a mindset to utilize it more frequently.

• “A few months ago, I hardly used it, but in the past few weeks, I’ve tried to integrate it into my daily routine, using it once or twice a day.” (P9S)

Some stated that they returned to their traditional methods of studying after using AI, as they felt more comfortable with this approach to learning.

• “Because honestly, I forgot. I’ve just resorted more to my own notes and my own resources.” (P13E)

Two participants did not use it, citing concerns about accuracy, potential undermining of their cognitive processes, and the risk of plagiarism.

• “I don’t use it very much at all, basically, but I think it could be useful.” (P17E)

Finally, in contrast, others explained that, given their new insights about AI and its potential limitations, they would use it less or more strategically, rather than being their “go-to” or primary source of information.

• “Since I’ve started to be involved in this project about AI, I’ve started to use AI less because it made me more cautious about the potential mistakes that AI makes. So, in reality, my use of AI has decreased” (P3E)

There was an overlap between this theme, primarily related to the motivation and the goals of using AI, and the “Benefits of AI” theme.

### Benefits of AI

The perceived benefits of AI highlight its growing role in medical education as a versatile learning aid. Students reported that AI improves their ability to manage the demanding workload of medical school by providing concise, well-organized information, instant feedback, and a non-judgmental environment for exploring concepts.

***Efficiency.*** A recurring benefit mentioned by students was the speed with which AI delivers information, saving valuable time compared to traditional study methods.

• *“Mostly the speed in getting a response. In a book, you have to read and analyze yourself to get the information, but with* ChatGPT *, it takes much less time, and the response is more specific and concrete to what I need.” (P4S)*

***Organized and concise information.*** Many students appreciated AI’s ability to present information in a neat, well-organized format, making it easier to comprehend and incorporate into their study notes.

• “It also sorts the information really neat and orderly so I can transfer it to notes.” (P13E)

***Personalization.*** AI was also described as a supplemental tool that fills gaps in their education when other resources, such as professors, are not readily available.

• “In the end, it’s an extra help. In the end, the professors are there, but they’re not available 24/7. So, if you’re unclear about something, or maybe the professor didn’t explain it well, you check it with it, and you get an idea. Then, you can finish it off with your notes or by going to the professor.” (P10S)

***Non-Judgmental learning environment.*** A significant advantage of AI noted by several students was its non-judgmental nature, which allowed them to ask any question without fear of being perceived as ignorant. This aspect of AI use created a safe space for learning and exploration, encouraging students to engage more actively with learning material.

• “It’s convenient, concise, and you can ask anything. Your professor might think you’re stupid, but the AI won’t judge you. You can have an explanation, and then if it says something like, ‘Oh, the mitochondria,’ you can ask, ‘What’s mitochondria?’ You can ask whatever you want, even you can say, ‘Explain this in caveman terms,’ which you can’t tell your professor, so it’s comfortable as well.” (P20E)

### Concerns and limitations of AI

While students reported the benefits of using AI in their medical studies, they also expressed significant concerns and identified limitations. These included issues related to the reliability of AI-generated information, its potential to stifle creativity, and the risk of over-reliance, which could hinder the development of critical thinking and independent learning skills.

***Reliability.*** A repeatedly stated concern from students was the reliability of AI in studying, and they were concerned that they were using a tool that could potentially teach them misinformation.

• “It confuses concepts, wrong words, or things that don’t even exist.” (P16E)
• “The negatives are that it’s not always right and it can influence your learning and understanding,” (P19E)
• “It has fooled me a few times with the wrong answers.” (P20E)
• “Yes, it makes mistakes, but it’s not like you should use it as if it were sacred text, but as a tool.” (P14S)
• *“Be careful because it’s not always right, but it thinks it is. Always rely on the class and the teacher because they’re the ones examining you. You can’t hold anyone accountable but yourself even if* ChatGPT *told me this.” (P20E)*

***Too general or too specific.*** While using AI to learn, students have voiced that the answers AI generates can be too robust or insufficient to answer their questions.

• “I just want to have a specific answer and then it tells me this long, long kind of a story. I don’t want to read it all.” (P1E)

***Undermine creativity and work ethic.*** Some students reported that using AI could impede their creative or critical thinking or hinder teamwork.

• “It can reduce creativity and critical thinking if you rely on it too much. It’s important to use your own mind and not become too dependent on AI.” (P9S)
• “One concern is that it can limit development and teamwork. For example, if everyone in a meeting relied on AI to generate different viewpoints, we might all end up with the same perspectives, reducing diversity in thought. It’s important to use AI consciously and be aware of its limitations.” (P9S)

***Over-reliance and excessive use.*** Finally, students expressed concerns regarding their cognitive development and the impact of AI on repeated use without fully developing the ability to learn and reason.

• *“I have to put in effort into learning, and I don’t know if I would learn in the long term using* ChatGPT *.” (P17E)*
• “You don’t want to over-rely on using AI because I’ve known a lot of people who have basically let AI run their academic life, and I just don’t want to lose my ability to research on my own.” (P8E)

### Ethics of AI use

Ethical considerations were a significant concern among students, particularly regarding plagiarism, work ethic, and preserving human qualities such as empathy and compassion in medical practice. While students acknowledged AI’s potential to support learning and decision-making, they emphasized the importance of maintaining human oversight and responsibility in educational and clinical settings.

***Plagiarism.*** Students expressed ethical concerns when using AI to learn. The most obvious and commonly discussed was using AI to generate text or write essays as their work.

• “There is, you know, plagiarism. It would be easier to pass some exams depending on where you are if you can use it. You can use it to cheat, and then you’re going to treat people with questionable knowledge.” (P18E)

***Work ethic.*** Others stated they were concerned that one’s accountability to learning could be compromised and lead to indolence.

• “Work ethic-wise it definitely contributes to laziness.” (P13E)

***Loss of empathy and compassion (human touch).*** As future doctors, students’ concerns were also that medicine could be changed for the worse and that we could lose the power of the human encounter.

• *“I personally think medicine is more than just giving an answer to a patient. I don’t see* ChatGPT *giving a hand to a terminal patient or just comforting people.” (P17E)*
• “Medicine isn’t just about information that you need to know, it’s also about interacting with the patient. And that’s something a machine can’t do. The most important part of being a doctor, so to speak, could be taken away. You should use AI to complement your profession, but it shouldn’t replace your profession.” (P11S)

***Human responsibility.*** Finally, another critical concern was the need for human oversight in decision-making when using AI in clinical settings. Students expressed apprehension about doctors potentially over-relying on AI to make clinical decisions without fully understanding its limitations, emphasizing that doctors should always be responsible for patient care, regardless of AI input.

• “But what does worry me is when we don’t teach doctors or future doctors how to use AI. In the future they might use it incorrectly. That’s what worries me, not the AI per se, but the use doctors give it. That’s the principal concern I have.” (P16E)
• “Doctors should ultimately be making their own choices, and if they decide to take the opinion of an AI as part of that, they still hold responsibility for whatever action they take, not the artificial intelligence” (P7E)

### Advice on AI use in medical education

Students offered practical advice to their peers, professors, and curriculum developers regarding AI’s responsible and effective use in medical education. Their advice emphasized the importance of using AI as a supportive tool, maintaining academic integrity, and incorporating AI instruction into the curriculum in a structured and meaningful way.

***Advice for peers.*** Students were asked to share the advice they would give their peers about using AI. Students encouraged their peers to use AI in their studies, but to be cautious about its output and not use it to the detriment of their learning.

• “I would tell them to first study and try to understand and then use it as a tool.” (P1E)
• *“It’s a really helpful platform to learn, but to always double-check first of all. I would recommend you and your teacher being the primary tool and your notes in class and then going to* ChatGPT *or any other AI resource as a secondary tool.” (P13E)*
• “Don’t use it to cheat. It’s very convenient and makes life easier, but in the long term, it’s going to harm you. Use it to prepare extra materials and learn more about some questions you have because it can be more efficient and saves you time.” (P18E)

***Advice for professors.*** Students were asked to share their advice on how professors could effectively utilize AI. Students encouraged professors to use AI in their teaching, to do so responsibly, and to be transparent about their use to facilitate effective teaching.

• “Professors should use AI in ways they would want others to use it. They need to be aware of the impact of their actions and use AI responsibly. For example, if students don’t understand a topic, professors can use AI to create additional study materials that could be beneficial.” (P9S)
• “Teachers should find ways to use AI to help study or make the course less heavy for us. If they find ways to help us, why not share those ways? Like the professor did in class the first semester. They told us about it, maybe not with the intention of helping us, but he surely did with me. He helped me out.” (P16E)

***Advice for curriculum development.*** Finally, students were positive and enthusiastic about integrating AI into the curriculum when asked whether AI should be included and how it could be included.

• “We have to start building both curriculum programs and sort of boundaries to get the best out of AI without risking our knowledge and the quality of our work for technology.” (P8E)
• “I would integrate it into classes because it’s going to be part of our lives. The sooner we learn the advantages, disadvantages, and how to use it properly, the better.” (P4S)
• “It is a tool and we as humans we have to use what we create and not demonize things. I would implement it in the curriculum.” (P16E)

### Attitudes toward and understanding of AI

This theme explores students’ sentiments toward AI, revealing a spectrum of attitudes that range from enthusiasm and curiosity to skepticism and uncertainty about its role in medical education and future clinical practice. Additionally, it examines students’ varying levels of understanding and familiarity with AI tools. While some students were optimistic about AI’s potential to transform learning and healthcare, others highlighted the need for proper instruction and critical engagement to avoid misuse and over-reliance.

• “I think we have to know and learn how to use AI without taking the reflection part out of the learning process.” (P1E)
• “In general, in today’s society, people don’t want to think. People want things instantly without spending time and energy doing things.” (P1E)

They also commented on how they think AI will impact medicine and their future roles as doctors.

• ” I’ve read that in areas like radiology, it could replace humans, but there will always be a need for medical supervision. In pathology, it could help count cells, but the final diagnosis will always be made by a doctor. It could speed up the public health system and improve emergency responses by monitoring vital signs.” (P2S)
• “It’s definitely going to change the profession. Some people say it’s going to replace doctors, but I don’t believe it. It’s hard to predict how medicine is going to be influenced exactly, but I guess over the years, the influence of AI is going to increase in all fields.” (P18E)
• “I don’t think it should ever overrule or overtake the position medical experts have.” (P8E)

### Participation in the project

This theme relates to the experiences of the students in the *AI UNAV Student Collaborative Team.* Their involvement in the project enhanced their understanding of AI and influenced their behavior and attitudes toward its use. By actively contributing to the research and curriculum design process, students gained a deeper appreciation for the complexities of AI, including its limitations, ethical considerations, and practical applications in medical education. This experience has empowered them as stakeholders in shaping how AI is integrated into their learning environment.

• “Since I’ve started to be involved in this project about AI, I’ve started to use AI less because it made me more cautious about the potential mistakes that AI makes. So, in reality, my use of AI has decreased.” (P3E)
• *“Be mindful of what you’re using it for. Remember that AI is constantly learning from your inputs. If you use it irresponsibly, like sharing confidential information or negative messages, it can have harmful effects. Know when and how to use it properly.” (P9S*)
• “Understand how it works so you can get the best out of AI and be aware that, I mean, for many people, it works like magic, but it’s a really complex process that because of its complexity, it’s prone to flaws.” (P8E)

## Discussion

This study focuses on first-year medical students at the point of entry into medical training, a formative transition when study strategies, help-seeking habits, and epistemic trust in information sources are particularly malleable. ^41^^–^^43^ Examining this window matters as early decisions about where to seek explanations, how to verify information, and when to rely on peers versus tools can set a trajectory that reverberates across preclinical learning and later. ^44^^,^^45^ From this vantage point, our analysis reveals how newcomers initially position generative AI as a supplement to traditional resources and how these positions evolve over the first months of medical school.

Across our findings and two recent qualitative studies with medical students in Sri Lanka and India, students consistently framed LLMs as being convenient, time-saving tools for clarifying complex concepts, producing concise explanations, and structuring studying. ^46^^,^^47^ These benefits were tempered by concerns about variable accuracy and over-reliance that demand verification and critical appraisal. Our contribution evaluates how these dynamics manifest at the beginning of medical school. In our cohort, 18/20 students reported regular or occasional use, most encountering LLMs on arrival and learning about them primarily through peers through horizontal diffusion. Several students described tapering their AI use as they adapted to a conventional study routine, suggesting an initial “bridge” function for AI rather than a stable dependency.

Comparatively, Sri Lankan students (from mixed year levels) highlighted social-educational costs, reduced collaboration and classroom engagement, along with ambivalent faculty messaging. ^46^ In comparison, Indian students (from mixed year levels) described a broader task repertoire (MCQ generation, drafting assignments, proofreading, and quick clinical vignettes. ^47^ Our first-year students instead highlighted AI as a “non-judgmental tutor,” valued for its iterative, personalized explanations and feedback on notes, alongside explicit ethical concerns (such as plagiarism and erosion of effort). Students who participated in our co-design process articulated more calibrated and skeptical views, aligning with evidence that structured, curriculum-embedded instruction improves AI knowledge and promotes safer practices. ^48^

In addition, students encouraged peers to utilize AI as a supplementary resource, advised professors to model responsible AI use, and advocated for curricula that integrate technical, ethical, and professional considerations to ensure students are prepared for its responsible application in medical practice. ^6^^,^^12^^,^^49^ Lastly, we found that students emphasized the importance of AI enhancing clinical practice without replacing empathy and personal interaction, underscoring the need for guidelines on the ethical use of AI in both educational and clinical settings. ^50^

Our findings reveal a key paradox in the modern medical student experience. This is the pursuit of academic efficiency through AI, which often conflicts with a deep concern for professional and ethical responsibility. This tension reflects the human-centered principle at the heart of the AAMC’s 2025 Principles for Responsible AI, which states that human judgment must be the ultimate decision-maker. ^51^ Participant narratives in the study strongly align with this principle, indicating that students see AI not as a replacement for their intellect but as a tool that demands careful ethical oversight. However, this desire for usefulness is balanced by the risk of “automation bias”. The World Health Organization warns that excessive reliance on LLMs may weaken clinical reasoning, a concern shared by our participants, who expressed concern about potential cognitive deskilling. ^52^ Ultimately, these findings highlight a critical need for “AI literacy” suggested by Triola and Rodman. ^53^ Our study indicates that students are actively seeking a structured, curriculum-based approach to better understand how to use AI in learning and in their future as clinicians.

There is mounting evidence of an urgent need for AI instruction to be incorporated into medical education curricula. ^9^^,^^10^^,^^54^ Understanding how students utilize AI early in their education provides a solid foundation for determining how AI instruction can be effectively integrated into medical curricula.

### Limitations

Several limitations are recognized. Convenience sampling may have introduced selection bias, as participants might have had stronger opinions or more experience with AI, potentially skewing the results. Moreover, half of the participants were from the *AI UNAV Student Collaborative Team.* However, including some students who use it less frequently and the broad spectrum of opinions expressed indicates that the study captured varying views and experiences. Researcher bias is another limitation, as several members of the research team had a special interest in AI. The potential for this was recognized from the outset of the study, and specific reflexivity strategies were integrated in the analysis and write-up process. Moreover, the research team included members with concerns and even skepticism about the use of AI in medical education, thereby providing additional balance. The members of the *AI UNAV Student Collaborative Team* who participated as researchers in this qualitative study – five of the twenty students – did not participate as interviewees, thereby reducing the bias. The reliance on self-reported data is subject to social desirability and recall biases, which could influence the responses. To mitigate the limitation of having novice researchers and interviewers participate in the study, we provided upfront training on qualitative methods, analysis, and interview techniques. We also included an experienced researcher in all interviews and had experienced researchers lead the coding and thematic analyses. Moreover, coding and theme identification were done by consensus.

## Conclusion

Our findings reveal that first-year medical students are, to varying degrees and in different ways, using AI and, largely, adopting generative AI as an adjunct for clarification and study organization. They recognize issues related to accuracy and integrity. As new study habits form upon entry to medical school, this study provides an evidence base for introducing AI instruction early and sustaining it throughout the curriculum. Students expressed enthusiasm for formal AI literacy education, emphasizing the importance of critical thinking, ethical use, and professional responsibility. Additionally, students requested longitudinal instruction in AI literacy, which included guidance on leveraging AI in clinical settings. Lastly, co-design participation was linked to more calibrated and skeptical practices, suggesting that structured guidance at the entry of medical school can shape safer AI use trajectories. The results of our study could support a future exploratory sequential design program of research on this topic, where the insights gained from the qualitative study inform the development of a larger survey.

## Ethics approval and consent to participate

The University of Navarra Research Ethics Committee reviewed and approved the study protocol (2024.084 mod1). All participants were informed of the study’s purpose, and written informed consent was obtained at the beginning of each interview. All participants agreed to the audio recording, either verbally or in writing. Confidentiality was warranted through anonymization. Participants had the right to withdraw at any time. All methods were conducted in accordance with relevant guidelines and regulations as outlined in the Declaration of Helsinki.

## Data Availability

In the case of this study, deidentified transcripts could contain specific examples, and open sharing would threaten participants’ confidentiality. Data can be obtained from the authors upon reasonable request, on a case-by-case basis, at jsimoni@unav.es. This request would include a thorough description of use, credentials of individuals getting access, confirmation of IRB approval for research, and our own IRB review and approval for the secondary use of the data. Supplementary materials (interview guide and Standards for Reporting Qualitative Research checklist) are available as extended data at *Supplementary material supporting the publication of “The Use of AI by First-Year Medical Students: A Qualitative Study of Perspectives, Usage, and Recommendations”.* https://doi.org/10.6084/m9.figshare.30287389, openly available on Figshare, under the terms of the Creative Commons Attribution 4.0 International license (CC-BY 4.0). This research followed the SRQR checklist for reporting qualitative research, which is available as part of the supplementary materials for this study.
