# Supplementary material for: AI Use or Non-use by First-Year Medical Students: A Qualitative Study of Perspectives, Usage, and Recommendations
Source: MedEdPublish (2016). 2026 Feb 18;15:226. Originally published 2025 Oct 27. [Version 2] doi: 10.12688/mep.21319.2 (PMC12980083; doi:10.12688/mep.21319.2)
Supplement: Peer review history

# Reviewer report: Reviewer response for version 2

**DOI:** 10.21956/mep.23037.r45441

**Published:** 2026-03-19

**Authors:** Helena Filipe

**Affiliations:** 1 Independent Researcher, Lisbon, Portugal

The opportunity to review this engaging article is appreciated. The authors explore the role of Artificial Intelligence (AI) in Health Professions Education. Their research focuses on the experiences and perceptions of first-year medical students, employing a qualitative investigation methodology. The article is generally well written and organised, with a particularly thorough approach to the methodology section, offering detailed explanations and descriptions.

 The abstract encapsulates the main points of the article and encourages readers to further explore the content.

 The authors demonstrate a clear commitment to achieving their overarching research objectives, which are planned to be addressed through several steps.

 In this version of the manuscript, the results section reflects considerable effort to respond to the reviewers´ feedback. Some of the queries and comments may have been influenced by cultural perspectives, but the authors have made a concerted effort to articulate their aims. The research team, which includes experienced faculty members, strives to advance scholarship (also by teaching) and student competencies illuminated by the CanMeds framework. The discussion section builds upon the results and introduces new opportunities for further research, with limitations addressed thoughtfully. The conclusion remains concise and true to the findings and reflections. The study adheres to ethical standards, as demonstrated throughout.

 Including students in this line of research is highly important. Their perspectives, experiences, and needs are expected to be shared with less or no academic bias and their involvement may inform curriculum development in meaningful ways. As first-year students in their early stages of professional identity formation, their flexibility is highlighted by the authors. Combining this malleability with their critical thinking and of the of senior members makes sense.

 Examining the correspondence between reviewers and authors offers meaningful educational perspectives on how varied personal backgrounds may influence the review process, particularly in situations involving multiple stakeholders and complex issues. The authors responded comprehensively and systematically, addressing comments respectfully and effectively.

 Limitations are acknowledged and do not undermine the significance of the research, especially given the rapidly evolving and complex nature of AI in Health Professions Education (HPE).

 In summary, this research is part of a stepwise approach to addressing a contemporary and relevant topic, the chosen methodology is explained transparently, the results gain from well-designed tables, and the discussion offers a thorough reflection.

 The paper aligns well with the scope of MedEdPublish, will attract interest from the wider HPE community, and represents a valuable contribution to the field, warranting indexing.

Have any limitations of the research been acknowledged?

Yes

Is the work clearly and accurately presented and does it cite the current literature?

Yes

If applicable, is the statistical analysis and its interpretation appropriate?

Yes

Are all the source data underlying the results available to ensure full reproducibility?

Partly

Is the study design appropriate and is the work technically sound?

Partly

Are the conclusions drawn adequately supported by the results?

Yes

Are sufficient details of methods and analysis provided to allow replication by others?

Yes

Reviewer Expertise:

Continuing Professional Development, Social Learning, Simulation Based Education, Online Education

I confirm that I have read this submission and believe that I have an appropriate level of expertise to confirm that it is of an acceptable scientific standard.

# Reviewer report: Reviewer response for version 2

**DOI:** 10.21956/mep.23037.r45420

**Published:** 2026-03-06

**Authors:** Waqar Naqvi

**Affiliations:** 1 Datta Meghe Institute, Wardha, Maharashtra, India

Thank you to the authors for the careful revisions and detailed responses to the previous review comments. The revised manuscript has improved in several areas, particularly in clarifying the sampling approach, expanding the methodological description, and strengthening the discussion of limitations and researcher positionality.

 The study provides an exploratory qualitative investigation into how first-year medical students engage with AI tools in their learning. The methodological approach using semi-structured interviews and reflexive thematic analysis is appropriate for exploring a relatively new and evolving phenomenon in medical education. The description of data collection, coding procedures, and consensus-based theme development is sufficiently detailed to allow readers to understand how the findings were generated.

 Some methodological limitations remain, particularly the relatively high proportion of participants drawn from the AI UNAV Student Collaborative Team, which may influence attitudes toward AI. However, this limitation is now explicitly acknowledged in the manuscript, and the authors provide appropriate contextualization. Additionally, while the decision not to provide a strict definition of “AI” may introduce variability in interpretation, the manuscript now illustrates the specific tools students referred to (e.g., ChatGPT and related platforms), which helps readers understand the context of participants’ responses.

 Overall, the manuscript presents a transparent and coherent qualitative study. While the findings should be interpreted within the context of a single institution and a small exploratory sample, the paper offers useful insights into how early medical students are beginning to integrate generative AI tools into their learning strategies and how they perceive the associated ethical and educational implications.

 I therefore consider the manuscript scientifically sound and suitable for indexing, provided readers interpret the findings within the acknowledged limitations.

Have any limitations of the research been acknowledged?

Partly

Is the work clearly and accurately presented and does it cite the current literature?

Yes

If applicable, is the statistical analysis and its interpretation appropriate?

Not applicable

Are all the source data underlying the results available to ensure full reproducibility?

Partly

Is the study design appropriate and is the work technically sound?

Yes

Are the conclusions drawn adequately supported by the results?

Yes

Are sufficient details of methods and analysis provided to allow replication by others?

Partly

Reviewer Expertise:

Medical Education, Health Professions Education, Artificial Intelligence in Education, Digital Health

I confirm that I have read this submission and believe that I have an appropriate level of expertise to confirm that it is of an acceptable scientific standard.

# Reviewer report: Reviewer response for version 2

**DOI:** 10.21956/mep.23037.r45418

**Published:** 2026-02-28

**Authors:** Alice Kam

**Affiliations:** 1 University of Toronto, Toronto, Canada

Thank you for addressing earlier concerns.  Authors placed all the bias concerns in the limitation sections.  Yet the study would not be improved regarding its methodology state. It may be helpful to consider perspective publications if biases are the limiting factor.

 The previous methodology was 2 non-AI users and rest with AI experiences from the author's lab.  In this revision, the number of participants changed.  The credibility of this study methodology is questionable at this stage, as research participant numbers should not be altered in a second revision.

Have any limitations of the research been acknowledged?

Partly

Is the work clearly and accurately presented and does it cite the current literature?

Partly

If applicable, is the statistical analysis and its interpretation appropriate?

Partly

Are all the source data underlying the results available to ensure full reproducibility?

No

Is the study design appropriate and is the work technically sound?

No

Are the conclusions drawn adequately supported by the results?

No

Are sufficient details of methods and analysis provided to allow replication by others?

No

Reviewer Expertise:

AI design

I confirm that I have read this submission and believe that I have an appropriate level of expertise to state that I do not consider it to be of an acceptable scientific standard, for reasons outlined above.

# Reviewer report: Reviewer response for version 1

**DOI:** 10.21956/mep.22831.r45188

**Published:** 2026-01-22

**Authors:** Alice Kam

**Affiliations:** 1 University of Toronto, Toronto, Canada

Tilte:

 The Use of AI by First-Year Medical Students: A Qualitative Study of Perspectives, Usage, and Recommendations

 -This title is clear, but this study is related to “the use of AI”.  In the method section, it mentioned about non-AI users.  Perhaps, adding: “a comparison study”.  The title needs to reflect to the content of the manuscript.

 Abstract:

 The abstract is clear and easy to understand; however, this study is a “qualitative study was conducted using semi-structured interviews to explore the experiences, attitudes, and perceptions of first-year students regarding AI”. Why would the authors include non-users “two were non-users”.  Non-users won’t be able to answer the “experience” question.  Do you want to compare users versus non-users? If you would like a comparison study, you need a sample size of more than 2.

 Background:

 The study highlights the needs of having guidance on AI technology and application in UGME.  It then go into their proposed way to understand the needs: “These include the study reported in this paper, a scoping review of the literature on AI in medical education (from 2022 to 2024), a Delphi process to identify the competencies and learning objectives to include in the medical curricula, and student-driven guidelines on the practical, professional, and ethical use of AI to support learning.”  The Delphi process is not well described in this manuscript.

 Research Study:

 Method is organized in study design, participants/settings, data collection, data analysis. However, it would be helpful to include the reasons for not defining AI: “We purposively chose not to define “AI” to allow students to discuss AI in a manner that reflected their personal experiences and perceptions.”  In the method, it discussed about reflection and it will be beneficial to describe how does this reflection happen? (e.g., what strategies were used to support reflection?) 

 Results:

 The results may potentially be biased if involving research team participants: “This theme relates to the experiences of the students in the *AI UNAV Student Collaborative Team*.” I would suggest further explanation of how the bias is mitigated in this study.

 Discussion:

 Discussion highlights a few key elements, such as urgent needs for responsible AI use.  The discussion will be further strengthened with current literature on responsible AI use.

 Conclusion:

 ”Students expressed enthusiasm for formal AI literacy education, emphasizing the importance of critical thinking, ethical use, and professional responsibility.” The recommendation is “Additionally, students requested longitudinal instruction in AI literacy, which included guidance on leveraging AI in clinical settings.” These are few interesting points that can be further explained in the discussion section to build a stronger conclusion.

 Unfortunately, this manuscript cannot be accepted with the current status based on the above.

Have any limitations of the research been acknowledged?

Partly

Is the work clearly and accurately presented and does it cite the current literature?

Partly

If applicable, is the statistical analysis and its interpretation appropriate?

Partly

Are all the source data underlying the results available to ensure full reproducibility?

No

Is the study design appropriate and is the work technically sound?

No

Are the conclusions drawn adequately supported by the results?

No

Are sufficient details of methods and analysis provided to allow replication by others?

No

Reviewer Expertise:

AI design

I confirm that I have read this submission and believe that I have an appropriate level of expertise to state that I do not consider it to be of an acceptable scientific standard, for reasons outlined above.

# Response

**Published:** 2026-02-04

**Authors:** Jennifer Simoni

**Affiliations:** Faculty of Medicine, Universidad de Navarra, Pamplona, Spain

Thank you for taking the time to review our manuscript. It is much appreciated. Below are our responses to the points raised in your review.

**1. Regarding including non-users and the title** As we explained to Reviewer 3, the term “Use” includes non-users, and this was done a priori and was included in the semi-structured questions. Although non-users cannot respond to the “experience” question, they can certainly explain why they have not used it, which also provides important insights. We have therefore changed the title to read “AI use or non-use by First-Year Medical Students: A Qualitative Study of Perspectives, Usage, and Recommendations”. We have also made it clearer in the stated study goals and in the Methods that we used the term “Use” broadly to also explore why some students may not be using it or

 using it to a lesser extent.  

**2.  Regarding describing the Delphi process** The Delphi process is not well described in this manuscript because it is not part of this study. No Delphi process was used in this qualitative study. It is a separate study that has been reported separately and is currently under review. We mention this here to provide context, i.e., that the interview study we report herein is part of a larger study program that included a scoping literature review (Simoni et al., 2025 - BMC Medical Education 25, 1609) and two activities using a Delphi approach.  

**3. Regarding not defining AI and the reflection process ** Please see responses to reviewers #2 and #3, as this is addressed there.  

**4.** **Regarding the bias of the research team participants** as we explain in our manuscript, the students who were part of the research team involved in the qualitative study, i.e., those who conducted the interviews under supervision, were not interviewed. As we share in our manuscript, another aspect of this work was to promote scholarship among medical students and to involve them in a co-design process for integrating AI into the curriculum. It would defeat the goal of achieving this if all students involved in the project were excluded. Therefore, we deliberately did not include them as interviewees (participants). To make this point clearer, we have added the following explanation at the end of the Introduction section: “Some of the students are interested in AI, while others are more interested in developing their scholarship skills, as one of the goals of the overall initiative is to nurture scholarship amongst students with a special interest in this area.” In addition, we have also added a line in the Limitations section stating: “Researcher bias is another limitation, as several members of the research team had a special interest in AI. The potential for this was recognized from the outset of the study, and specific reflexivity strategies were integrated in the analysis and write-up process. Moreover, the research team included members who had concerns and even skepticism related to the use of AI in medical education, thereby providing additional balance. The members of the *AI UNAV Students Collaborative Team* who participated as researchers in this qualitative study - five of the twenty students - did not participate as interviewees, thereby reducing the bias”.

**5. Regarding strengthening the discussion with current literature on the responsible use of AI.**

 Thank you for the comment about including details in the Discussion section on “current literature on responsible AI use”. We have added this to the Discussion section. Thank you again, JS, JP, EM, and the research team

# Reviewer report: Reviewer response for version 1

**DOI:** 10.21956/mep.22831.r45187

**Published:** 2026-01-21

**Authors:** Ken Masters

**Affiliations:** 1 Sultan Qaboos University, Muscat, Oman

**Your Report**

 Please provide a full report, expanding on your answers to the questions above. In particular, if you answered “no” or “partly” to any of the questions, please give constructive and specific details as to how the authors can address any criticisms. Please indicate clearly which points must be addressed to make the article scientifically sound. ( *50 words min.*)

 The paper sets out to report on a qualitative study on the use of AI by first-year medical students at the institution. 

 While the paper is an interesting read, it suffers from major problems in methodology, and these cannot be corrected at this stage.  In summary, the problems are: (1) including non-users in a study of usage, (2) not defining AI, (3) including a disproportionate number of students from the *AI UNAV Student Collaborative Team.  *These are described in more detail below.

  

1. “The study described herein aims to explore, in more depth, how first-year medical students currently use AI in their undergraduate education, including their motivations, methods of use, and overall experiences.”  This was a study of _users_.  Non-users should have been screened and excluded from the study interviews.  Including non-users in a study of users invalidates a significant portion of the data  (for example, there would be the same problem if the study set out to discover the views of medical students, and then included the views of non-medical students).  The parameters of the study sample are set by the title and the aim; the researchers went outside those parameters, and have damaged their data.   (If their non-use was discovered only during the interview, then the interview should have ended, and their data excluded from the study. )

 

1. “We purposively chose not to define “AI” to allow students to discuss AI in a manner that reflected their personal experiences and perceptions. Our goal was to capture the full range of student interactions with AI tools, regardless of the specific technologies or platforms they used.”   This is a major problem with the paper.  A definition and / or description of the term “AI” was essential.  Without that, students baseline understanding conflicts with each other, and so students’ use and opinions of AI are, unfortunately, of little value, as the interviewers could be using it in one sense, and the interviewees in a completely different sense; or two interviewees could appear to be in agreement with each other, but actually be completely opposed because their understanding and use of the term “AI” is completely different.  Not having this this defition or description further damages the data.

 

1. “Of the 20 students (12 female and 8 male), 10 were part of *AI UNAV Student Collaborative Team*, and the other 10 were not involved in the AI initiative.”  Unless the *AI UNAV Student Collaborative Team *has a massive number of students (approximately 50% of the student body), this is a major problem of bias.  By its very nature, students who are part of the *AI UNAV Student Collaborative Team *are biased towards the use of AI, and such a heavy representation of the interview population will result in a non-representation of the student population.   At best, the researchers should have had numbers proportionate to the student population, and only members who were not part of the research group or authors should have been interviewed.  As it stands, however, the sample is not representative of the student body.  Although the authors have mentioned this as a limitation, it is more than a “limitation” – it is a problem that damages the results beyond repair.  Mentioning a problem in a limitation, unfortunately, does not remove that problem.

In addition, the conclusions in the Abstract are too broad.  “Students are early adopters of AI, using it in various ways, and wish to utilize it effectively and ethically. Medical curricula should ensure early AI literacy.”  This was a single study at a single institution.  Any conclusions apply to that institution only.

 As a result of the major problems, unfortunately, the paper cannot be recommended for indexing.

Have any limitations of the research been acknowledged?

Yes

Is the work clearly and accurately presented and does it cite the current literature?

Yes

If applicable, is the statistical analysis and its interpretation appropriate?

No

Are all the source data underlying the results available to ensure full reproducibility?

Yes

Is the study design appropriate and is the work technically sound?

No

Are the conclusions drawn adequately supported by the results?

No

Are sufficient details of methods and analysis provided to allow replication by others?

Yes

Reviewer Expertise:

NA

I confirm that I have read this submission and believe that I have an appropriate level of expertise to state that I do not consider it to be of an acceptable scientific standard, for reasons outlined above.

# Response

**Published:** 2026-02-04

**Authors:** Jennifer Simoni

**Affiliations:** Faculty of Medicine, Universidad de Navarra, Pamplona, Spain

We thank you for your review and comments. However, we respectfully disagree with the reasons given for recommending rejection of the manuscript. Below is our point-by-point response.     

**(1) Regarding including non-users in a study of usage.** The study goal was to explore why and how medical students are using or not using AI to support their learning. We applied the term “use” to include varying levels of use, including non-use. We wanted to explore why and how students are using AI, and if not, why not. To this end, our interview questions, established before the interviews began, included questions relevant to non-users of AI. Therefore, we stand by our decision to include non-users. A clinical analogy could be the view of a community on vaccinations. It would be important to understand why some would not want to use it. We recognize that we could have been more explicit in our use of the word “use” and our desire to include non-users. We have therefore modified the research goal statement in the revised manuscript to read “The study described herein aims to explore, in more depth, why and how first-year medical students currently use AI, or don’t use it, in their medical training, including their motivations for using it or not using it, methods of use, and overall experiences”   We have also adjusted the title to read “AI use or non-use by First-Year Medical Students: A Qualitative Study of Perspectives, Usage, and Recommendations”, and made this more explicit in the Participants subsection. 

**(2) Regarding not defining AI.**   We respectfully disagree with the reviewer. In phenomenology, when exploring a phenomenon, it is acceptable to use a broad term to examine how people interpret it differently.  A clinical analogy would be to explore the concept of compassion and what it means to different patients and care providers. If we were to provide a definition of what that is up front, we would limit the exploration of that phenomenon. We deliberately asked them what they were using AI for (they were using for AI) and how they were using it (they were using it), to explore what they meant by it. In our study, we found that participants often referred to large language models when discussing AI and confirmed that they had some understanding of what AI is. Our impression is that this is a generation that is more informed about the digital world, including AI, than we may give them credit for.

**(3) Regarding including a disproportionate number of students from the AI UNAV Collaborative Team.**

 The reviewer is of the opinion that a disproportionate number of students from the AI UNAV Student Collaborative Team were interviewed. We ensured a balanced representation amongst the interviewees of those in the *AI UNAV Student Collaborative Team* and those not. In qualitative research, the goal is to reach thematic sufficiency, which is done by interviewing until saturation of codes and themes is reached. This occurred in both groups, and we therefore feel confident that the experiences of both groups (students in the *AI UNAV Student CollaborativeTeam* and students unrelated to the Team) were adequately captured. Moreover, not all the students in the *AI UNAV Student Collaborative Team* joined because of an interest in AI. Some joined because they were interested in developing their scholarship skills. Therefore, the assumption that all students in the AI *UNAV Student Collaborative Team *were there because of a special interest in AI is not correct. In addition, to mitigate bias, the students who were involved in the interviews and the analysis for this study were not interviewed (they were not study participants). We revised the Methods section of the original manuscript to state more clearly: “The five students who were part of the research team in this qualitative study did not participate as interviewees.”  Additionally, some students were not using AI much, and some were skeptical of it.  Qualitative research explores a phenomenon in depth; in this case, it includes students who use AI and those who use it little or not at all. All these students have a valid contribution to make in terms of their experiences, and we captured these adequately.   With reference to representing the student body, this is not a quantitative study examining generalizability.

**(4) In regard to the abstract being too broad.**

 The conclusions in the abstract of our revised manuscript now read “We found that students in the medical school we studied are early adopters of AI, using it in various ways, and wish to utilize it effectively and ethically. The findings of this study align with other studies in other jurisdictions that call for early AI literacy.”  

 Thank you again,

 JS, JP, EM, and the research team

# Reviewer report: Reviewer response for version 1

**DOI:** 10.21956/mep.22831.r45170

**Published:** 2026-01-16

**Authors:** Waqar Naqvi

**Affiliations:** 1 Datta Meghe Institute, Wardha, Maharashtra, India

This is a timely, well-conducted, and thoughtfully analyzed qualitative study that makes a valuable contribution to the literature on AI in medical education. Only minor clarifications related to methods, limitations, and data availability are recommended. The article is scientifically sound and suitable for indexing following minor revisions.

 1. Greater clarity on how thematic sufficiency was operationalized and assessed would be helpful.

 2. The interview guide is referenced as extended data; briefly summarizing its core domains in the main text could improve transparency.

 3. More detail on how disagreements during coding were resolved (beyond consensus discussions) would strengthen methodological clarity.

 4. The potential influence of participants’ prior interest in AI (particularly those involved in the AI collaborative team).

 5. Limits to transferability beyond similar institutional and cultural contexts.

Have any limitations of the research been acknowledged?

Partly

Is the work clearly and accurately presented and does it cite the current literature?

Yes

If applicable, is the statistical analysis and its interpretation appropriate?

Not applicable

Are all the source data underlying the results available to ensure full reproducibility?

Partly

Is the study design appropriate and is the work technically sound?

Yes

Are the conclusions drawn adequately supported by the results?

Yes

Are sufficient details of methods and analysis provided to allow replication by others?

Partly

Reviewer Expertise:

Medical Education, Health Professions Education, Artificial Intelligence in Education, Digital Health

I confirm that I have read this submission and believe that I have an appropriate level of expertise to confirm that it is of an acceptable scientific standard, however I have significant reservations, as outlined above.

# Response

**Published:** 2026-02-04

**Authors:** Jennifer Simoni

**Affiliations:** Faculty of Medicine, Universidad de Navarra, Pamplona, Spain

Thank you for your review and comments. They are much appreciated. We are encouraged by the overall assessment of it being a good and useful study.

 1. Regarding greater clarity on how thematic sufficiency was operationalized and assessed would be helpful. In our manuscript, we used the term “thematic sufficiency” to refer to saturation. In the revised manuscript, we have changed it to “saturation”, which is a more commonly used term (Methods: Participants and setting). Saturation (or “thematic sufficiency”) was identified through the process of iterative to-and-fro coding. As we explain in our manuscript (Methods: Data analysis), we adhered to the six phases of thematic analysis as described by Braun and Clarke. This includes familiarizing with the data, generating initial codes, identifying themes, reviewing themes, and defining and naming themes. As described, the processes of coding, searching for themes, and reviewing themes were conducted iteratively and by consensus during regular research team meetings.  We undertook peer debriefing (where we engaged in regular discussions as a research team, iteratively reviewing and refining the codes and themes, and intentionally applying reflexivity (for reasons we have also described in our manuscript). This iterative, consensus-based, to-and-fro process allowed us to identify when saturation had been reached and ensured adequate exploration of the data to identify codes and themes. We regularly and repeatedly asked ourselves whether we had fully coded all relevant data, whether the codes were applicable and appropriate, whether we had fully and appropriately captured and grouped related codes into potential themes and whether the themes we identified were representative of the data, whether the themes had been fully refined to ensure coherence and relevance, whether the themes were each supported by sufficient data excerpts, and that no key aspects of the data (and codes and themes) had been overlooked. In looking back, we believe all this is aligned with a checklist recently described by Ahmad et al (Ahmed SK, et al. Using thematic analysis in qualitative research. J Med Surg and Public Health 2025; 6, 100198 https://doi.org/10.1016/j.glmedi.2025.100198). To address the reviewer’s request, we have rephrased part of the text and have added this information in the revised manuscript (Methods section: Data analysis).

 2. The interview guide is referenced as extended data; briefly summarizing its core domains in the main text could improve transparency.

 In the original manuscript, the key semi-structured interview questions were included in the Methods section under Data Collection. In the revised manuscript, we have made this more straightforward by writing “These questions included ….” after referring to the questions presented to the participants.

 3. More detail on how disagreements during coding were resolved (beyond consensus discussions) would strengthen methodological clarity.

   We started from the position that all input was respected, so all points of view were heard. When there was disagreement, a discussion was held to ensure the different views were voiced and understood. From that discussion, we reached a consensus solution that reflected their thinking, or, if their perspective changed, we adjusted accordingly. We had assumed that readers would understand what is meant by reaching consensus; however, to make this even clearer in the revised manuscript, we have now added the explanation provided above (Methods section: Data Analysis). 

 4. The potential influence of participants’ prior interest in AI (particularly those involved in the AI collaborative team). The goal of our study was to explore the use of AI (or not) by medical students. It was therefore appropriate to include students who were using AI and were interested in AI. We also included interviewees who were not using AI to capture their thoughts and experiences, as aligned with the research question. If, however, the reviewer is referring to the research team members' interest in AI (noting that some were using it and others were not), we agree that such an interest could introduce bias into the analysis. For that reason, we deliberately adopted reflexivity strategies that were described in our original manuscript.  However, we realized we may not have made this point clear enough in the Limitations section, and have added the following statements:

 “Researcher bias is another limitation, as several members of the research team had a special interest in AI. The potential for this was recognized from the outset of the study, and specific reflexivity strategies were integrated into the analysis and write-up processes. Moreover, the research team also included members with concerns and even skepticism about the use of AI in medical education, thereby providing additional balance.”    

 5. Limits to transferability beyond similar institutional and cultural contexts. With respect to transferability, we believe the manuscript provides sufficient information and description of the context and research methods, allowing readers to interpret the results, undertake similar research, and determine whether the results are applicable to their setting. We respectfully believe that the modifications made in the revised manuscript provide the information that may have previously been missing, thereby enabling transferability. 

 Thank you again,

 JS, JP, EM, and the research team

# Reviewer report: Reviewer response for version 1

**DOI:** 10.21956/mep.22831.r44513

**Published:** 2025-12-24

**Authors:** Suriyaarachchige Nishan Silva

**Affiliations:** 1 Ochre health, Sydney, Australia

This is a qualitative study aimed to explore the experiences, attitudes and perceptions of first year medical students regarding AI. The study was based on interviewing 20 conveniently selected medical students using semi structured interviews. 18 have used AI and 2 hasn't. This I feel is not the ideal method to realise the mentioned objective. Either this could have been a survey or (if still remaining as a qualitative design) the selection of the sample could have been a purposeful one. Nevertheless, the topic and even more the findings are relevant, important and note-worthy.  The manner in which the results are presented is more like aligning quotes under themes to support them rather than depicting how themes were derived from the interview quotes/the content mentioned in the interview transcripts. Other than these few points that could have been improved, this is a good study.

Have any limitations of the research been acknowledged?

Yes

Is the work clearly and accurately presented and does it cite the current literature?

Yes

If applicable, is the statistical analysis and its interpretation appropriate?

Not applicable

Are all the source data underlying the results available to ensure full reproducibility?

Partly

Is the study design appropriate and is the work technically sound?

Partly

Are the conclusions drawn adequately supported by the results?

Yes

Are sufficient details of methods and analysis provided to allow replication by others?

Yes

Reviewer Expertise:

Health Informatics, Health Professions Education, AI, Primary Care

I confirm that I have read this submission and believe that I have an appropriate level of expertise to confirm that it is of an acceptable scientific standard.

# Response

**Published:** 2026-01-13

**Authors:** Jennifer Simoni

**Affiliations:** Faculty of Medicine, Universidad de Navarra, Pamplona, Spain

Thank you for your review and comments. They are much appreciated. We are encouraged by the overall assessment of it being a good and useful study. We believe that our research question was best addressed with a qualitative study. Our study goal was to explore, in more depth, why and how students are using AI for medical learning. Qualitative research methods are best suited to this. A survey would have been more appropriate if our goal were to assess the extent to which students use AI. However, with respect to a survey, our team feels that the results of our study support a future exploratory sequential design program of research on this topic, ****where the insights from the qualitative study inform the development of a larger survey more targeted toward generalizability.  Regarding purposive sampling instead of convenience sampling, we considered it at first but decided against it due to concerns about introducing selection bias. Having said that, at the end, the participants included were, we believe, representative of the mixture of experiences and use of AI by students, as they included students using AI extensively, others using it to a lesser degree, and some not using it at all.  We believe the results are presented appropriately as themes and, as is usual practice in qualitative research, are supported with illustrative quotes. The themes were identified by way of an iterative thematic analysis that is explained in the Methods section of the manuscript Thanks again.

 JS, JP, and EM

# Response

**Published:** 2026-02-04

**Authors:** Jennifer Simoni

**Affiliations:** Faculty of Medicine, Universidad de Navarra, Pamplona, Spain

Additional comments

 Thank you for your review and comments. They are much appreciated. We are encouraged by the overall assessment of it being a good and useful study. We believe that our research question was best addressed with a qualitative study. Our study aimed to explore in greater depth why and how students use AI for medical learning. Qualitative research methods are best suited to this. A survey would have been more appropriate if our goal were to assess how much students use AI. However, regarding the survey, our team believes the results of our study support a future exploratory sequential design program of research on this topic, in which the insights from the qualitative study inform the development of a larger, more generalizable survey. Regarding the sampling approach, we used a combination of convenience and purposive sampling to ensure that the views of students at various levels of AI use were included and explored. We have made this clearer in our revised manuscript (Methods Section: Participants and settings). Finally, we believe the results are presented appropriately as themes and, as is usual practice in qualitative research, are supported with illustrative quotes. The themes were identified through an iterative thematic analysis, as explained and further clarified in the revised manuscript (Methods Section: Data analysis).

 Thank you again,

 JS, JP, EM, and the research team
